# Epigenetic DNA methylation of Zbtb7b regulates the population of double-positive CD4^+^CD8^+^ T cells in ulcerative colitis

**DOI:** 10.1186/s12967-022-03477-6

**Published:** 2022-06-27

**Authors:** Hao-ming Xu, Jing Xu, Mei-feng Yang, Yu-jie Liang, Quan-zhou Peng, Yuan Zhang, Cheng-mei Tian, Yu-qiang Nie, Li-sheng Wang, Jun Yao, De-feng Li

**Affiliations:** 1 Department of Gastroenterology and Hepatology, Guangzhou Digestive Disease Center, Guangzhou First People’s Hospital, School of Medicine, South China University of Technology, Yuexiu District, No. 1, Panfu Road, Guangzhou, 510180 Guangdong China; 2 Department of Hematology, Yantian District People’s Hospital, Shenzhen, 518020 Guangdong China; 3 Shenzhen Kangning Hospital, Shenzhen, 518020 Guangdong China; 4 Department of Pathology, Shenzhen People’s Hospital (The Second Clinical Medical College, Jinan University, The First Affiliated Hospital, Southern University of Science and Technology), Shenzhen, 518020 Guangdong China; 5 Department of Medical Administration, Huizhou Institute of Occupational Diseases Control and Prevention, Huizhou, 516000 Guangdong China; 6 Department of Emergency, Shenzhen People’s Hospital (The Second Clinical Medical College, Jinan University, The First Affiliated Hospital, Southern University of Science and Technology), Shenzhen, 518020 Guangdong China; 7 Department of Gastroenterology, Shenzhen People’s Hospital (The Second Clinical Medical College, Jinan University, The First Affiliated Hospital, Southern University of Science and Technology), Luohu District, No. 1017, Dongmen North Road, Shenzhen, 518020 Guangdong China

**Keywords:** Ulcerative colitis, Zbtb7b, DNA methylation, Colitis model

## Abstract

**Background and aims:**

Ulcerative colitis (UC) is a heterogeneous disorder with complex pathogenesis. Therefore, in the present study, we aimed to assess genome-wide DNA methylation changes associated explicitly with the pathogenesis of UC.

**Methods:**

DNA methylation changes were identified by comparing UC tissues with healthy controls (HCs) from the GEO databases. The candidate genes were obtained and verified in clinical samples. Moreover, the underlying molecular mechanism related to Zbtb7b in the pathogenesis of UC was explored using the dextran sodium sulfate (DSS)-induced colitis model.

**Results:**

Bioinformatic analysis from GEO databases confirmed that Zbtb7b, known as Th-inducing POZ-Kruppel factor (ThPOK), was demethylated in UC tissues. Then, we demonstrated that Zbtb7b was in a hypo-methylation pattern through the DSS-induced colitis model (*P* = 0.0357), whereas the expression of Zbtb7b at the mRNA and protein levels was significantly up-regulated in the inflamed colonic tissues of UC patients (qRT-PCR, WB, IHC: *P* < 0.0001, *P* = 0.0079, *P* < 0.0001) and DSS-induced colitis model (qRT-PCR, WB, IHC: *P* < 0.0001, *P* = 0.0045, *P* = 0.0004). Moreover, the expression of Zbtb7b was positively associated with the degree of UC activity. Mechanically, over-expression of Zbtb7b might activate the maturation of CD4^+^T cells (FCM, IF: *P* = 0.0240, *P* = 0.0003) and repress the differentiation of double-positive CD4^+^CD8^+^T (DP CD4^+^CD8^+^T) cells (FCM, IF: *P* = 0.0247, *P* = 0.0118), contributing to the production of inflammatory cytokines, such as TNF-α (*P* = 0.0005, *P* = 0.0005), IL-17 (*P* = 0.0014, *P* = 0.0381), and IFN-γ (*P* = 0.0016, *P* = 0.0042), in the serum and colonic tissue of DSS-induced colitis model.

**Conclusions:**

Epigenetic DNA hypo-methylation of Zbtb7b activated the maturation of CD4^+^T cells and repressed the differentiation of DP CD4^+^CD8^+^ T cells, resulting in the production of inflammatory cytokines and colonic inflammation in UC. Therefore, Zbtb7b might be a diagnostic and therapeutic biomarker for UC, and hypo-methylation might affect the biological function of Zbtb7b.

**Supplementary Information:**

The online version contains supplementary material available at 10.1186/s12967-022-03477-6.

## Introduction

As an idiopathic inflammatory disorder, ulcerative colitis (UC) is one of the primary inflammatory bowel diseases (IBDs) [1]. UC extends contiguously from the proximal colon and is mainly involved in the colonic mucosa and submucosa, contributing to ulceration, bleeding, fulminant colitis, and colorectal cancer [2]. Epidemiological evidence suggests that the incidence of UC has steadily increased worldwide, with up 286 per 100,000 people in the USA [3]. Although the incidence of UC is lower in China compared with Western countries, the number of UC patients is still continuously increasing [4].

Although it has been reported that genetic susceptibility, host immune system, and environmental factors, as well as intestinal microbiota, may play vital roles in the pathogenesis of UC, the current knowledge on the pathophysiology of UC is still poorly understood [5, 6]. Epigenetic mechanisms, such as DNA modification, histone protein modification, and chromatin modification, play an essential role in the pathogenesis of UC [7, 8]. Meanwhile, an increasing number of studies have reported that DNA methylation pattern plays a regulatory role in gene transcription, which may initiate and maintain intestinal mucosal inflammation by mediating the interplay between these predisposing genes and external and internal factors in UC [7, 9–12]. Furthermore, it has been demonstrated that the DNA methylation change in mucosal biopsies and peripheral blood is detected in both adults and children with UC [13, 14]. Of note, investigating the landscape of DNA methylation may contribute to a comprehensive understanding of UC pathogenesis.

Therefore, we first explored the DNA methylation changes in the present study by comparing UC samples with healthy control (HC) samples via Gene Expression Omnibus (GEO). We found that the hypo-methylation of Zbtb7b was detected in UC mucosal biopsies, which also prompted us to focus on Zbtb7b. Subsequently, we found that the expression of Zbtb7b was significantly up-regulated in UC samples, and it was associated with the degree of UC activity. Previous studies have shown that the expressions of CD4 and CD8 on T cells are usually mutually exclusive and tightly regulated by Zbtb7b, and double-positive (DP) CD4^+^CD8^+^ T cells have been identified in intestinal tissue [15–17]. Furthermore, we validated that Zbtb7b activated CD4^+^T cells and repressed CD4^+^ CD8^+^T cells in the inflamed colonic tissues of UC, which contributed to the colonic inflammation in the dextran sodium sulfate (DSS)-induced colitis model. Collectively, our current findings provided a new perspective on potential diagnostic biomarkers and therapeutic targets for UC.

## Methods

### DNA methylation profile from GEO datasets

The GSE27899 of methylation profiling was obtained from GEO databases, which compared 10 active UC samples with 10 HCs using the GPL8490 platform. In addition, the GSE1123, GSE38713, GSE48959, GSE59071, GSE87461, and GSE87466 of expression profiling were obtained from GEO databases. The raw data were normalized and analyzed by R software 4.1.0 (*limma* package). Differentially expressed genes (DEGs) were screened by classical t-test with the cutoff criteria of *p*-value < 0.05 and |fold change (FC)|> 1.0. In addition, Gene Ontology (GO) and Kyoto Encyclopedia of Genes and Genomes (KEGG) were conducted by the R cluster Profiler package. The immune cell infiltration, including B cells, natural killer (NK) cells, CD4^+^ T cell, CD8^+^ T cell, monocytes, and neutrophils, was compared between colonic tissue samples from UC patients and HCs. Moreover, R software 4.1.0 (*limma* package) was adopted to conduct cluster heatmap, correlation heatmap, and differential analysis.

### Patients and samples

Colonic biopsies were obtained from 15 active UC patients and 15 HCs in Shenzhen People's Hospital between April 2021 and July 2021. The tissue specimens were stored in liquid nitrogen for quantitative real-time PCR (qRT-PCR) and Western blotting (WB) analysis, while paraffin-embedded tissue specimens were used for immunohistochemistry (IHC) assay. Written informed consent was obtained from all participants. This study was approved by the Ethics Committee of Shenzhen People's Hospital (no. LL-KY-2021183), and experimental procedures complied with the Helsinki Declaration.

### Animals

A total of 15 male C57BL/6 mice were purchased from Guangdong Medical Laboratory Animal Center (GDMLAC; certificate number SYXK 2013-0002, Foshan, China). The mice were housed in a climate-controlled animal facility at 22 °C under a 12-h light/dark photoperiod and given free access to water and a standard rodent diet. The mice weighing 20–25 g (8–10 weeks old) were used in the experimental procedure. The experimental protocol was approved by the Animal Care Committee of Shenzhen People’s Hospital (no. LL-KY-2021183).

### DSS-induced colitis model

DSS was used to induce colitis as previously described [18]. Briefly, colitis was induced by 3% DSS in drinking water for 7 days. The status of the mice, such as body weight, stool consistency, and rectal bleeding, was recorded daily. In addition, the disease activity index (DAI) was assessed daily through the extent of body weight loss, stool hemoccult positivity or gross bleeding, and stool consistency as previously described [19]. After 7 days of the administration, blood was obtained through the retro-orbital vein, and serum was collected via centrifugation at 5000×*g* for 15 min and stored in liquid nitrogen for further analysis. Subsequently, the mice were sacrificed, the colons were dissected, and their length was determined. The colons were cut lengthwise along the mesentery and cleaned with normal saline, distal colons were fixed in 10% neutral buffered formalin overnight, some stored in liquid nitrogen for further use (qRT-PCR, WB, and ELISA), and some fresh tissues were directly used for flow cytometry (FCM).

### Hematoxylin–eosin (H&E) staining

The H&E staining was performed as previously described [20]. Briefly, the tissue sections were evaluated histopathologically by two independent evaluators in a blinded manner based on (a) the degrees of inflammation (0: no inflammatory infiltrate; 1: infiltrates in the lamina propria; 2: infiltrates in the submucosa; and 3: transmural infiltration), (b) ulceration (0: no ulceration; 1: one or two ulcers; 2: three or four ulcers; 3: more than four ulcers), (c) mucosal hyperplasia (0: normal; 1: slightly thickened mucosa with minimal fibrosis; 2: mucosal thickening with fibrous hyperplasia; 3: extensive mucosal thickening and fibrous hyperplasia or granulation), and (d) edema (0: none; 1: 0–30%; 2: 30–70%; 3: > 70%) (Microscope: Nikon E100/ Nikon DS-U3, Tokyo, Japan).

### Methylation-specific PCR (MSP)

Genomic DNA was extracted from colon samples using the HiPure Tissue DNA Mini Kit (Cat.: D3121-02, MAGEN, Guangzhou, China). The quantity of DNA was measured using NanoDrop 2000 (Thermo Scientific, Massachusetts, USA). Subsequently, sodium sulfite was added to the DNA samples, followed by purification using the reaction column with the EpiTect Bisulfite Kit (Cat.: 59104, QIAGEN, Duesseldorf, Germany). Finally, the acquired DNA samples were used for MSP. The primer sequences of Zbtb7b were as follows: 165 bp: 5′-GGGTTGGATTTAGGTTAGTGGTAC-3′ (M primer-F) and 5′-AACGAAAAAAACAAAAAAATCGTC-3′ (M primer-R); 166 bp: 5′-GGGTTGGATTTAGGTTAGTGGTAT-3′ (U primer-F) and 5′-AAACAAAAAAAACAAAAAAATCATC-3′ (U primer-R) (Sangon Biotech, Shanghai, China). PCR was carried out using PCR Master Mix (Cat.: DRR071A, TAKARA, Kyoto, Japan). Briefly, after an initial denaturation step at 95 °C for 5 min, amplifications were carried out with 40 cycles at a melting temperature of 94 °C for 15 s, an annealing temperature of 65 °C for 20 s, and an extension temperature of 72 °C for 30 s. The acquired PCR products were subjected to electrophoresis on 1% agarose gel and visualized under UV light [21]. The intensity of MSP was analyzed, the gray values of all bands were calculated through ImageJ software, and then the relative methylation percentage of the target gene (Zbtb7b) was determined using the equation as follows: [gray value of methylated (M) band/gray value of methylated (M) band + gray value of unmethylated (U) band] [22].

### RNA extraction and qRT-PCR

The RNA extraction and qRT-PCR were carried out as previously described [23]. The primers were listed as follows: Zbtb7b (human): 5′-CCTGTCTGCCACAAGATCATCC-3′ (forward) and 5′-GCATGTGGATCTTCAGCTTGTCG-3′ (reverse) (Sangon Biotech, China); GAPDH (human): 5′-CAAGAGCACAAGAGGAAGAGAG-3′ (forward) and, 5′-CTACATGGCAACTGTGAGGAG-3′ (reverse) (Sangon Biotech, China); Zbtb7b (mouse): 5′-CACACTGGTGAGAAGCCCTTTG-3′ (forward) and 5′-GTTCTCCTGTGTGCTTCCGCAT-3′ (reverse) (Sangon Biotech, China); GAPDH (mouse): 5′-TGCGACTTCAACAGCAACTC-3′ (forward) and, 5′-ATGTAGGCAATGAGGTCCAC-3′ (reverse) (Sangon Biotech, Shanghai, China). GAPDH was adopted as a housekeeping gene.

### WB analysis and IHC assay

WB analysis and IHC assay were carried out as previously described [24, 25]. Antibodies against ZBTB7B were purchased from Proteintech (Cat.:11341-1-AP, Wuhan, China). Antibodies against GAPDH were obtained from Affinity Biosciences (Cat.: AF7021, Jiangsu, China). In addition, goat anti-rabbit IgG and mouse/human ads-HRP were provided by Southern Biotech (Cat.: 4050-05A, Labama, USA). Antibody dilutions of ZBTB7B were 1:1000 for WB analysis and 1:100 for IHC assay. The relative gray value of WB was analyzed through ImageJ software, and then the relative gray value (relative expression) was calculated as follows: the gray value of the target protein (ZBTB7B)/the gray value of housekeeping protein (GAPDH). Two evaluators independently evaluated the sections in a blinded manner. Protein expression was assessed according to the extension and intensity of staining and scored as the extension of 0: 0–5%; 1: 5–25%; 2: 26–50%; 3: 51–75%; 4: 76–100% of cells stained positively and the staining intensity of 0: no staining; 1: weak staining; 2: moderate staining; 3: strong staining (Microscope: Nikon E100/Nikon DS-U3, Tokyo, Japan; 3D HISTECH Pannoramic MIDI, Budapest, Hungary).

### Enzyme-linked immunosorbent assay (ELISA)

The levels of TNF-α (Cat.: SEA133Mu), IL-17 (Cat.: SEA063Mu), and IFN-γ (Cat.: SEA049Mu) in the serum and colonic tissue were quantified using commercial ELISA kits according to the manufacturer’s instructions (Cloud-Clone, Wuhan, China).

### FCM

Sample processing: (a) The intestinal tissue was cut into small pieces with a scalpel or scissors to ensure no resistance when blowing by a 2-mL pipette; (b) the tissue was put on the shaking table and shaken at 80 rpm for 1 h; (c) during digestion, the cells were resuspended for several times; (d) the cell suspension was filtered using a 300-mesh screen, transferred to a new 15-mL centrifuge tube, washed with PBS, and then centrifuged at 400×*g* for 5 min; (e) the supernatant was discarded, and the pellet was washed twice with PBS and then centrifuged at 400×*g* for 5 min; (f) the supernatant was discarded again, and the pellet was resuspended in 1 mL flow buffer and transferred to the flow tube at 4 °C.

For FCM, the following fluorophore-labeled monoclonal antibodies (mAbs) were used: CD45-Alexa Fluor 532 (AF532), CD3-fluorescein isothiocyanate (FITC), CD4-peridinin-chlorophyll-protein (PerCP), and CD8a-Brilliant Violet 510 (BV510) (eBioscience, California, USA; Biolegend, California, USA). FCM was performed using the Cytek Aurora Full Spectrum Profiling Flow Cytometry (Cytek, California, USA), and SpectroFlo software was used for analysis (Cytek, California, USA). The antibodies of appropriately diluted concentrations were added to 100 μL heparin-processed tissue samples, followed by incubation at 4 °C for 30 min. Subsequently, the lymphocytes were gated as forward-scattered light and side-scattered light. Subsequent analysis was performed on these cell populations [26].

### Immunofluorescence (IF)

IF was performed as previously reported [25, 27]. Paraffin-embedded colonic sections (from five mice in each group) were dewaxed with dimethyl-benzene and hydrated through a graded series of ethanol. The sections were blocked with 3% BSA and then incubated with the first type of primary antibody (anti-CD4 rabbit pAb, Cat.: GB13064-2, Servicebio, Wuhan, China) at 4 °C overnight. After the sections were washed with TBST three times, they were incubated with the secondary antibody (HRP-conjugated goat anti-rabbit IgG, Cat.: GB23303, Servicebio, Wuhan, China) at room temperature for 50 min. After washing the sections with TBST three times, the sections were incubated with CY3-TSA (Cat.: G1223, Servicebio, Wuhan, China) at room temperature for 10 min in the dark. The sections were washed with TBST three times. Sections were placed in a repair box filled with citric acid antigen repair buffer (pH 6.0) and heated in a microwave. Sections were then incubated with the second type of primary antibody (anti-CD8 rabbit mAb, Cat.: GB13429, Servicebio, Wuhan, China) at 4 °C overnight. After washing the sections three times with TBST, sections were incubated with the secondary antibody (HRP-conjugated goat anti-rabbit IgG, Cat.: GB23303, Servicebio, Wuhan, China) at room temperature for 50 min. After washing the sections with TBST three times, sections were incubated with FITC-TSA (Cat.: G1222, Servicebio, Wuhan, China) at room temperature for 10 min in the dark. Sections were washed three times as above-mentioned, and nuclei were stained with DAPI for 10 min at room temperature. Finally, the sections were sealed with anti-fluorescence quenching sealant. Confocal microscopy (Nikon Eclipse C1/Nikon DS-U3, Tokyo, Japan) was used to examine the sections (DAPI emits blue light with an excitation wavelength of 330–380 nm and an emission wavelength of 420 nm; FITC emits green light with an excitation wavelength of 465–495 nm and an emission wavelength of 515–555 nm; CY3 emits red light with an excitation wavelength of 510–560 nm and an emission wavelength of 590 nm). The mean density was analyzed by ImageJ software.

### Statistical analysis

Continuous variables were summarized as mean ± standard deviation (SD)/standard error of the mean (SEM), and statistical analysis was performed using the SPSS 23.0 software package (SPSS Company, Chicago, USA). Differences between the two groups were analyzed using the Student’s t-test, while a two-way analysis of variance (ANOVA) was used to conduct multi-group comparisons. *P*-value < 0.05 was considered statistically significant.

## Results

### DNA methylation analysis

**Fig. 1:**
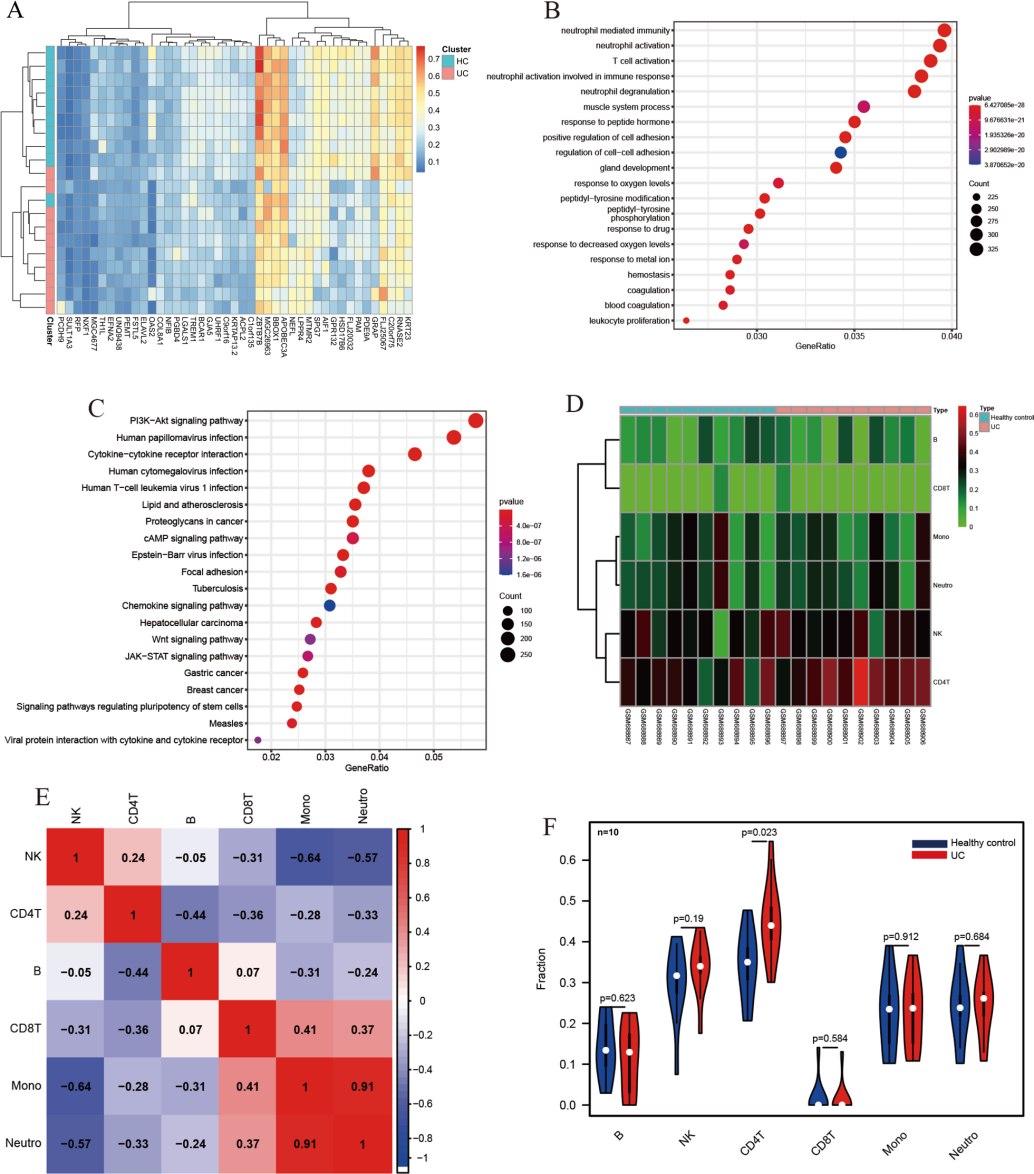
DNA methylation, enrichment analysis, and correlation analysis reveal the significant function related to Zbtb7b in UC patients and HCs. **A** Cluster heatmap of 48 differential DNA methylation acquired from GSE27899 (included 10 UC samples and 10 HCs), and 10 differential DNA hypermethylation and 38 differential DNA hypo-methylation in the UC samples. **B** Bubble chart of GO functional enrichment analysis showed the differentially methylated genes and enriched terms, which were mainly enriched in neutrophil-mediated immunity, neutrophil activation, and T cell activation. **C** Bubble chart of KEGG pathway analysis revealed differentially methylated genes and enriched pathways, which were enriched in the PI3K-Akt signaling pathway, human papillomavirus infection, and cytokine-cytokine receptor interaction. **D**, **E** Heat map and correlation heat map of immune cells in UC pathogenesis based on GSE27899, showing that immune cell infiltration played a vital role in UC. **F** Violin diagram of the proportion of six types of immune cells showed the difference in infiltration between HCs and UCs, illustrating that the proportion of CD4^+^ T cells was dramatically higher in UC samples compared with HC samples. *HC* healthy control, *UC* ulcerative colitis, *B* B cells, *NK* natural killer cells, *CD4T* CD4^+^ T cell, *CD8T* CD8^+^ T cells, *Mono* monocyte, *Neutro* neutrocyte

The DNA methylation profiles of GSE27899 were acquired from GEO databases, which included 10 UC samples and 10 HCs. There were 48 differential DNA methylations, including 10 differential DNA hypermethylation and 38 differential DNA hypo-methylation in the UC samples (Table 1; Fig. 1A). Figure 1A shows the methylation degree of Zbtb7b in HCs was more significant compared with the other genes, while it was significantly decreased in UC patients, suggesting that the hypo-methylation degree of Zbtb7b was related to the occurrence and development of UC. GO functional enrichment analysis showed that the differentially methylated genes were mainly enriched in neutrophil-mediated immunity, neutrophil activation, and T cell activation (Fig. 1B). The KEGG pathway analysis revealed that the differentially methylated genes were mainly enriched in the PI3K-Akt signaling pathway, human papillomavirus infection, and cytokine-cytokine receptor interaction (Fig. 1C). The landscape of immune cell infiltration was investigated by comparing UC patients with HCs, showing that immune cell infiltration played a vital role in the pathogenesis of UC. Figure 1D, F illustrate that the proportion of CD4^+^ T cells was dramatically higher in UC samples compared with HC samples. Moreover, the abundance of CD4^+^ T cells was positively associated with NK cells (Fig. 1E).

**Table 1:** The differential DNA methylation based on GSE27899

	Gene				
Hyper-methylation	SULT1A3	PCDH9	RFP	NXF1	FSTL5
	C10orf70	UNQ9438	PEMT	ELAVL2	CPT1A
Hypo-methylation	TH1L	SPINK2	EFNA2	NFIB	COL8A1
	PGBD4	KRTAP13-2	MGC4677	FKBP9L	C1orf135
	TREM1	NEFL	C9orf16	LPPR4	LGALS1
	ACPL2	UHRF1	MTMR2	GJA5	BCAR1
	PAM	PDE9A	FLJ25067	GPR132	OAS2
	FLJ23447	AIF1	FLJ20032	SPG7	C20orf75
	HSD17B6	BBOX1	KRT23	RNASE2	APOBEC3A
	MGC26963	GRAP	ZBTB7B

### DNA methylation and expression of Zbtb7b at the mRNA level in UC patients using bioinformatic analysis

Figure 1A shows that hypo-methylation of Zbtb7b was significantly observed in UC samples compared with HC samples. Zbtb7b, also known as Th-inducing POZ-Kruppel factor (ThPOK), is primarily identified as a transcription regulator in CD4^+^T cells. It has been reported that the ZBTB7B promotes the generation of CD4^+^T cells, which represses the differentiation of CD8^+^T cells [28–30]. We found that the proportion of CD4^+^T cells was dramatically higher in UC samples compared with HC samples (Fig. 1D, F).

**Fig. 2:**
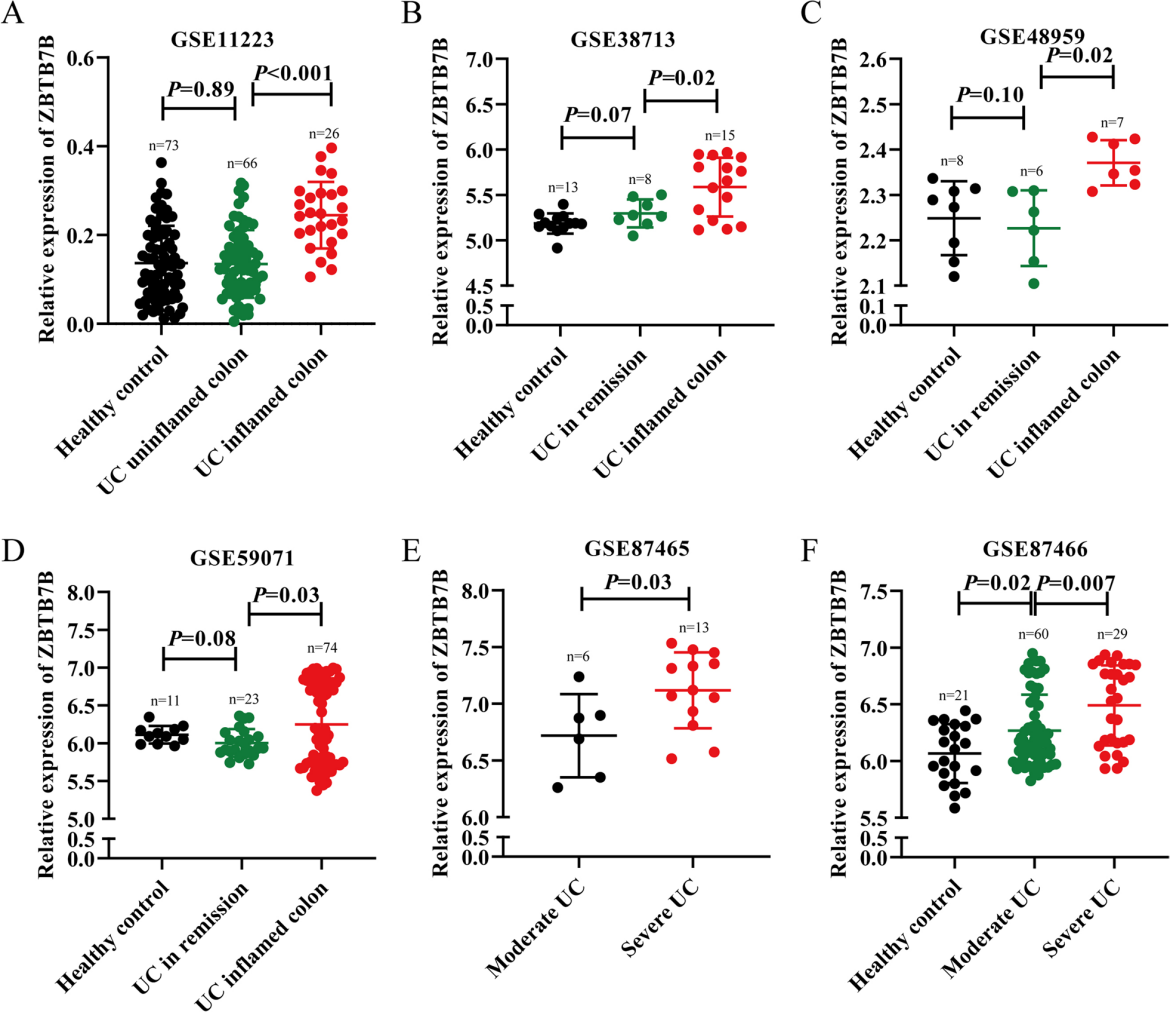
The expression of Zbtb7b in UC patients and HCs based on GEO databases. **A**–**F** Differential expression of Zbtb7b in GSE11223, GSE38713, GSE48959, GSE59071, GSE87465, and GSE87466. Six datasets contained 126 UC samples and 327 HC samples: inflamed colon UC samples, uninflamed colon UC samples, severe UC colon samples, moderate UC colon samples, and HC samples, which showed that Zbtb7b was significantly up-regulated in UC inflamed colon samples/severe UC colon samples compared with UC uninflamed colon samples/moderate UC colon samples and HC samples. *UC* ulcerative colitis

Therefore, the expression of Zbtb7b in UC was detected. The gene expression profile of GSE11223, GSE38713, GSE48959, GSE59071, GSE87465, and GSE87466 was acquired regarding UC from GEO databases. In dataset GSE11223, which contained 26 UC inflamed colon samples, 66 UC uninflamed colon samples, and 73 HC samples, we found that Zbtb7b was significantly up-regulated in UC inflamed colon samples compared with UC uninflamed colon samples and HC samples. In contrast, there was no significant difference in the expression of Zbtb7b between UC uninflamed colon samples and HC samples (Fig. 2A). In dataset GSE38713, which contained 15 UC inflamed colon samples, eight UC in remission colon samples, and 13 HC samples, we found that Zbtb7b was significantly up-regulated in UC inflamed colon samples compared with UC in remission colon samples and HC samples. At the same time, there was no significant difference between UC in remission colon samples and HC samples (Fig. 2B). Consistently, in dataset GSE48959, which contained seven UC inflamed colon samples, six UC in remission colon samples, and eight HC samples, we found that Zbtb7b was significantly over-expressed in UC inflamed colon samples compared with UC in remission colon samples and HC samples (Fig. 2C). Similarly, in dataset GSE59071, which contained 74 UC inflamed colon samples, 23 UC in remission colon samples, and 11 HC samples, we found that Zbtb7b was significantly elevated in UC inflamed colon samples compared with UC in remission colon samples and HC samples (Fig. 2D). In dataset GSE87465, which contained 13 severe UC colon samples and six moderate UC colon samples, we found that the expression of Zbtb7b was significantly greater in severe UC colon samples compared with moderate UC colon samples (Fig. 2E). In dataset GSE87466, which contained 29 severe UC colon samples, 60 moderate UC colon samples, and 21 HC samples, we found that Zbtb7b was significantly up-regulated in severe UC colon samples compared with moderate UC colon samples and HC samples. Meanwhile, a higher expression of Zbtb7b was detected in the moderate UC colon samples compared with HC samples (Fig. 2F).

### Protein and mRNA expression of Zbtb7b in UC patients

**Fig. 3:**
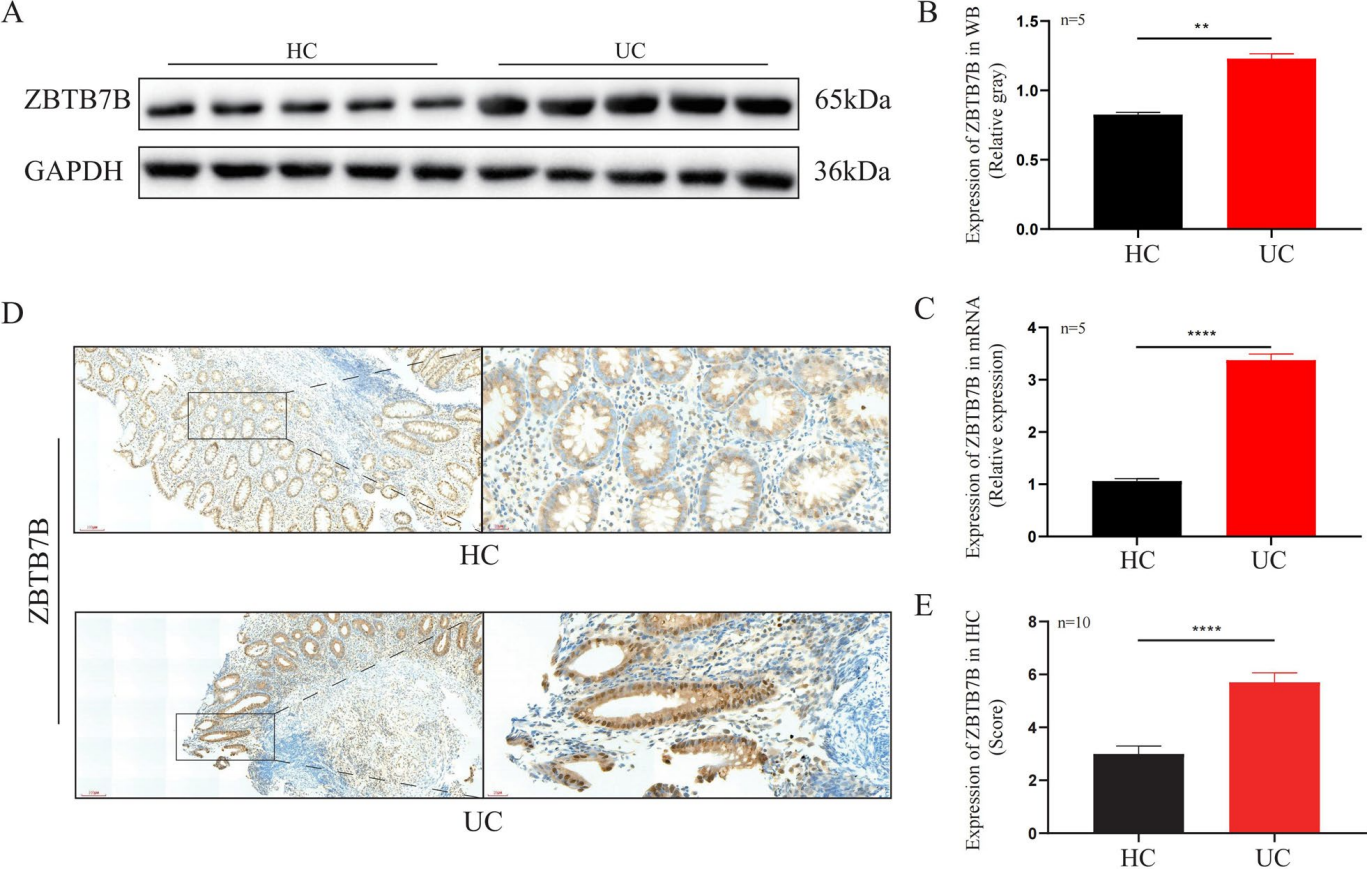
Protein and mRNA expression of Zbtb7b in UC patients and HCs. **A**, **B** WB and relative gray analysis of ZBTB7B protein in colonic tissue of five UC patients and five HCs. **C** Relative mRNA expression of Zbtb7b in colonic tissue of five UC patients and five HCs detected by qRT-PCR. **D**, **E** IHC and relative expression score of ZBTB7B protein in colonic tissue of 10 UC patients and 10 HCs. Data are representative images (scale bar: 100 μm and 20 μm) or expressed as the mean ± SEM of each group. **P < 0.01, ****P < 0.0001. *HC* healthy control, *UC* ulcerative colitis

The expression of Zbtb7b at the protein and mRNA levels was determined through WB, IHC, and qRT-PCR in 15 active UC colon samples and 15 HC samples (Patients’ information is listed in Additional file 3: Table S1). Five pairs of colonic biopsy tissues were used for WB (Fig. 3A, B) and qRT-PCR (Fig. 3C), while 10 pairs of paraffin-embedded colonic tissues were used for IHC analysis (Fig. 3D, E). It was shown that the expression of Zbtb7b at the mRNA level was significantly up-regulated in UC (*P* < 0.0001). In addition, WB and IHC showed that the expression of ZBTB7B at the protein level was significantly increased in UC (*P* = 0.0079, *P* < 0.0001).

### Establishment of DSS-induced colitis model

**Fig. 4:**
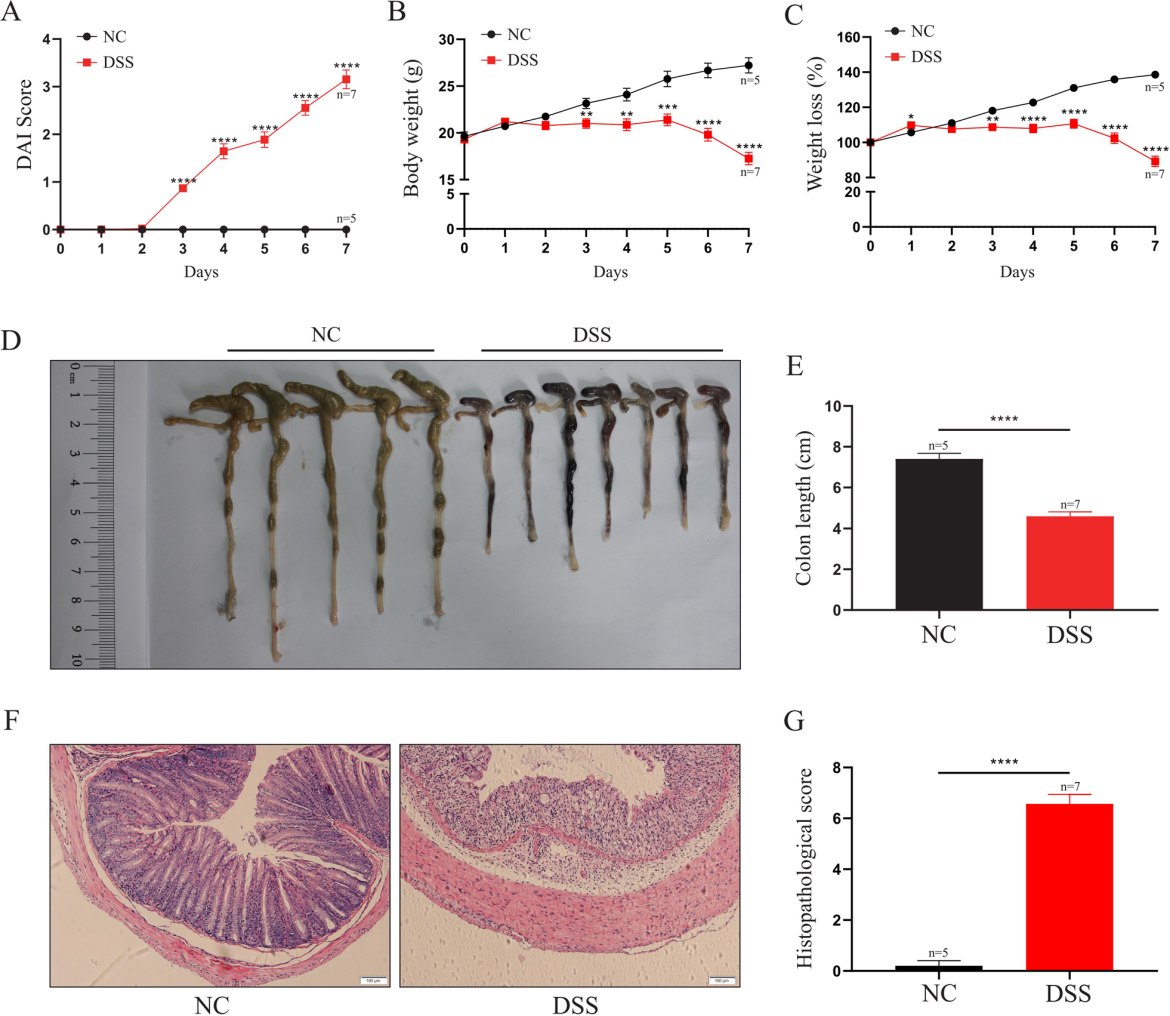
General indicators and colonic pathology of DSS-induced colitis mice and control mice. A total of 15 C57BL/6 mice were randomly divided into the NC group (n = 5) and the DSS group (n = 10, three mice died). DSS-induced colitis model was administered by 3% DSS for 7 days in the drinking water, and the dynamic process of colitis was monitored. **A**–**C** DAI score (**A**), body weight (**B**), and weight loss (**C**) were worsened significantly in the colitis group compared with the NC group. DAI score was significantly increased in a fluctuating manner in the DSS group, while the body weight and weight loss were decreased considerably from the 3rd day of DSS administration. **D**, **E** Colon length was significantly shortened in the colitis group compared with the NC group. **F**, **G** The histopathological changes: The NC group displayed clear colon mucosal structure, integrated epithelium, and ordered glands with enriched goblet cells, but without conspicuous inflammatory infiltrates in the lamina propria. In contrast, the DSS group exhibited severely damaged epithelium with a few epithelial cells, incomplete glands, and wide spreading of inflammatory infiltrates, which were the hallmarks of inflammatory colonic injury. Data are colonic macrograph, representative histopathological images (scale bar: 100 μm) or expressed as the mean ± SEM of each group. *P < 0.05, **P < 0.01, ***P < 0.001 ****P < 0.0001. *NC* normal control group, *DSS* DSS-induced colitis group

To further explore the underlying molecular mechanism related to Zbtb7b in the pathogenesis of UC, we established a DSS-induced colitis model in C57BL/6 mice by administering 3% DSS for 7 days in the drinking water, and the dynamic process of colitis was monitored. A total of 15 mice were randomly divided into two groups: the normal control group (NC group, n = 5) and the DSS-induced colitis group (DSS group, n = 10). Moreover, three mice in the DSS group died from severe diarrhea and bloody stool caused by experimental colitis before the end of the experiment. Figure 4A–C show that compared with the NC group, the DAI score was significantly increased in a fluctuating manner in colitis mice (DSS group), while the body weight and weight loss were decreased considerably from the 3rd day of DSS administration (all *P* < 0.05). Besides, the colon lengths (Fig. 4D, E) in the DSS group were significantly shorter compared with the NC group (*P* < 0.0001). Similarly, analysis of colonic tissues (*P* < 0.0001) revealed that the control mice displayed clear colon mucosal structure, integrated epithelium, and ordered glands with enriched goblet cells but without conspicuous inflammatory infiltrates in the lamina propria. In contrast, the mice in the DSS group exhibited severely damaged epithelium with a few epithelial cells, incomplete glands, and wide spreading of inflammatory infiltrates, which were the hallmarks of inflammatory colonic injury (Fig. 4F, G).

### Expression and hypo-methylation of Zbtb7b in mice with DSS-induced colitis

**Fig. 5:**
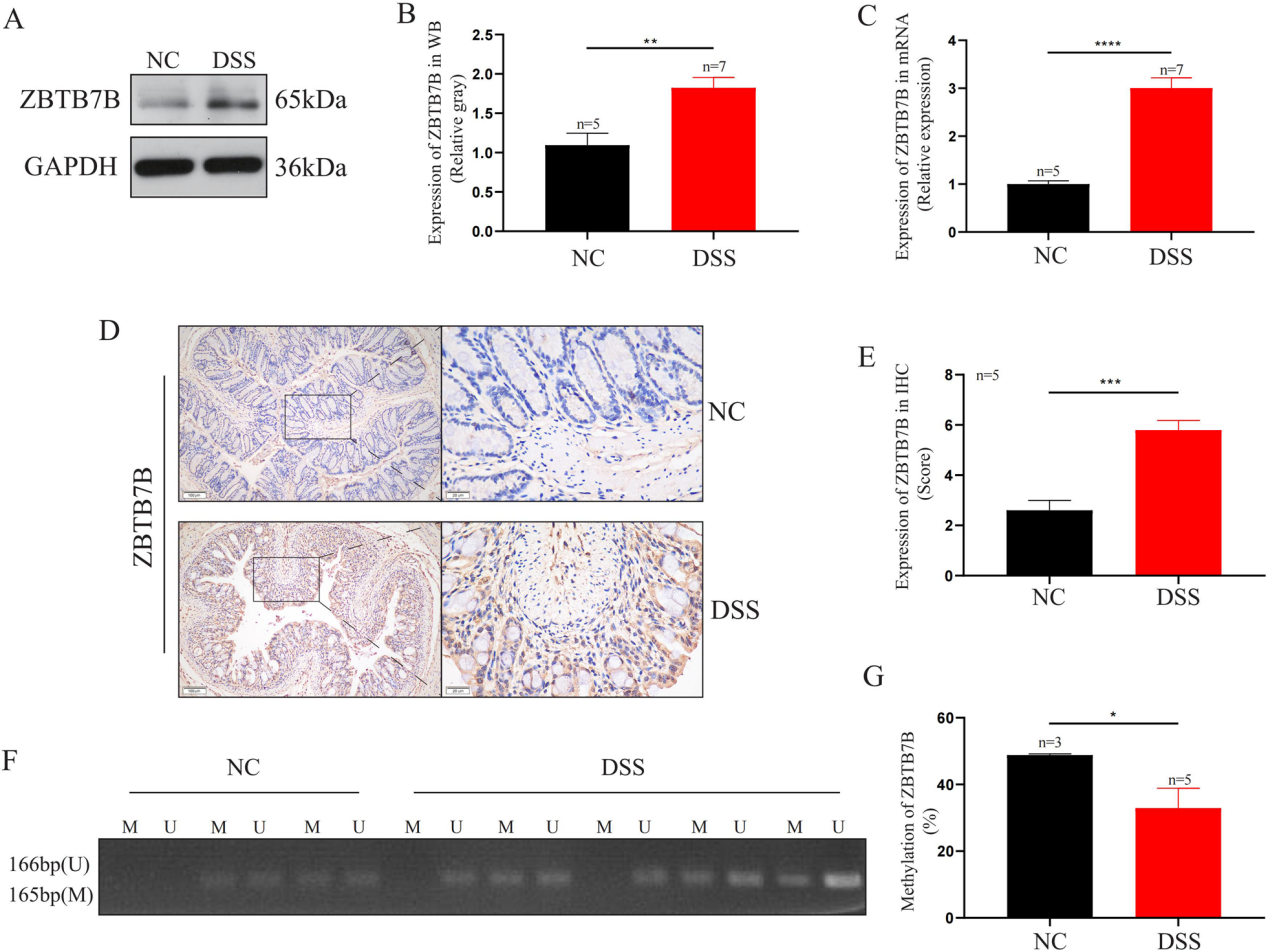
Expression and methylation of Zbtb7b in DSS-induced colitis mice and control mice. Colonic tissues of five control mice and seven colitis mice were used for WB and qRT-PCR, five pairs of paraffin-embedded colonic tissues were used for IHC analysis, while colonic tissues of three control mice and five colitis mice were used for MSP. **A**, **B** WB and relative gray analysis of ZBTB7B protein in colonic tissue of mice. **C** Relative mRNA expression of Zbtb7b in colonic tissue of mice detected by qRT-PCR. **D**, **E** IHC and relative expression score of ZBTB7B protein in colonic tissue of mice. **F**, **G** Methylation of Zbtb7b in colonic tissue of mice detected by MSP. Data are representative electrophoretic bands or expressed as the mean ± SEM of each group.*P < 0.05, **P < 0.01, ***P < 0.001, ****P < 0.0001. *NC* normal control group, *DSS* DSS-induced colitis group, *M* methylated, *U* unmethylated

Colonic tissues from five control mice and seven colitis mice were used for WB (Fig. 5A, B) and qRT-PCR (Fig. 5C), while five pairs of paraffin-embedded colonic tissues were used for IHC analysis (Fig. 5D, E). It was shown that the expression of Zbtb7b at the mRNA level was significantly up-regulated in DSS-induced colitis (*P* < 0.0001). In addition, WB and IHC showed that the expression of ZBTB7B at the protein level was significantly increased in DSS-induced colitis (*P* = 0.0045, *P* = 0.0004). Moreover, we found that the methylation level of Zbtb7b was significantly decreased (*P* = 0.0357) compared with the control mice (Fig. 5F, G). The complete WB results of all mice are shown in Additional file 1: Fig. S1.

### Zbtb7b represses the differentiation of CD4^+^CD8^+^T cells and promotes colonic inflammation in murine colitis

**Fig. 6:**
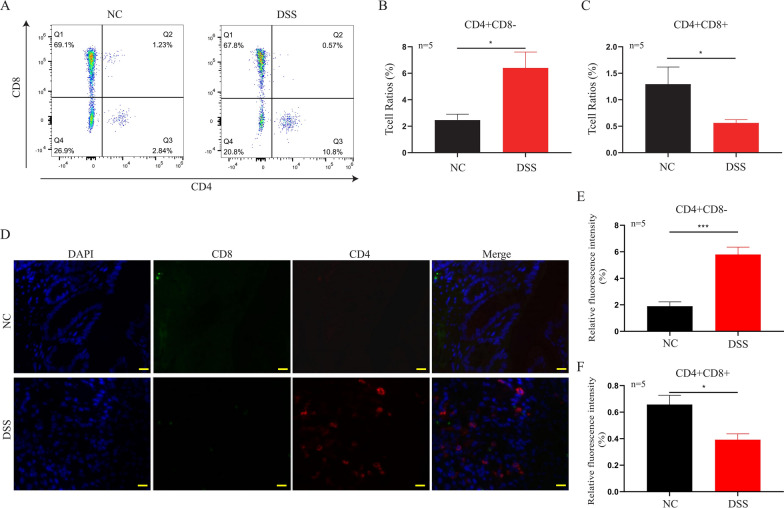
CD4^+^ T cells and DP CD4^+^CD8^+^ T cells in mouse intestinal tissue detected by FCM and IF. Colonic tissues of five pairs of mice were used for FCM and IF to detect the population of CD4^+^ T cells and DP CD4^+^ CD8^+^ T cells. **A**–**C** FCM showed that the population of CD4^+^T cells was increased, while the population of DP CD4^+^CD8^+^ T cells was decreased significantly in the DSS group compared with the NC group. **D**–**F** IF showed that the population of CD4^+^CD8^+^ T cells was significantly decreased, whereas the population of CD4^+^ T cells was increased dramatically in the DSS group compared with the NC group (Nucleus emitted blue light, CD8^+^ T cells emitted green light, while CD4^+^ T cells emitted red light). Data are representative FCM and IF images (scale bar: 20 μm) or expressed as the mean ± SEM of each group. *P < 0.05, ***P < 0.001. *NC* normal control group, *DSS* DSS-induced colitis group

**Fig. 7:**
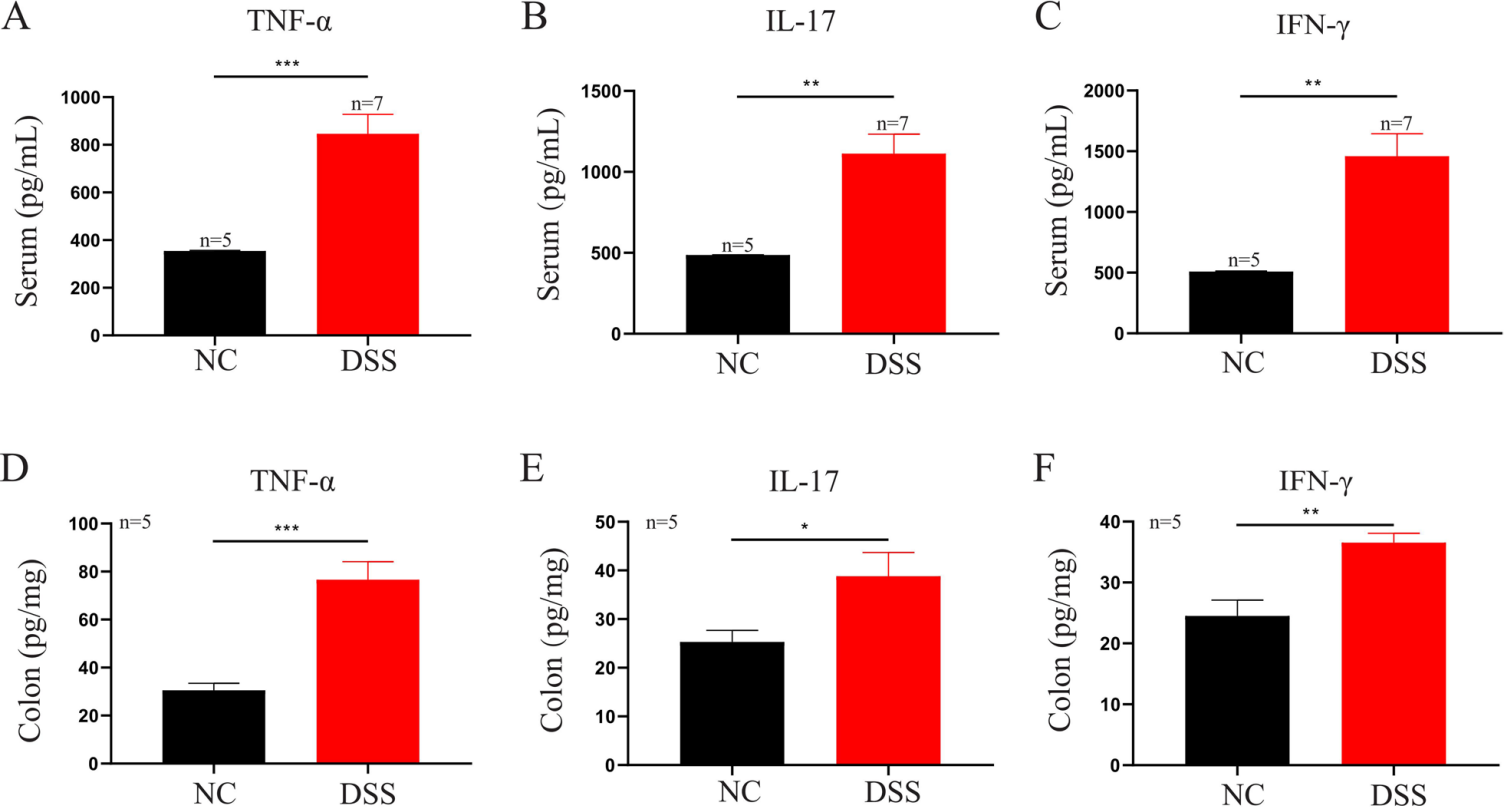
Serum and colonic inflammatory factor in mice. The serum samples of five control mice and seven colitis mice were used for analysis, while colonic tissues of five pairs of mice were used for inflammatory factor detection. **A**–**C** Serum levels of TNF-α, IL-17, and IFN-γ were detected by ELISA. **D**–**F** Colonic levels of TNF-α, IL-17, and IFN-γ were detected by ELISA. Data are represented as the mean ± SEM of each group. *P < 0.05, **P < 0.01, ***P < 0.001. *NC* normal control group, *DSS* DSS-induced colitis group

Through FCM, we found that the population of CD4^+^CD8^+^T cells (*P* = 0.0247) was significantly decreased, whereas the population of CD4^+^T cells (*P* = 0.0240) was increased dramatically in the DSS group compared with control mice (Fig. 6A–C). Specific steps of detecting CD4^+^/CD8^+^ T cells by FCM are shown in Additional file 2: Fig. S2. Similarly, through IF, we found that the population of CD4^+^CD8^+^ T cells (*P* = 0.0118) was significantly decreased, whereas the population of CD4^+^ T cells (*P* = 0.0003) was increased dramatically in the DSS group compared with control mice (Fig. 6D–F). A previous study [31] has shown that CD4^+^ T cells can significantly elevate the expressions of inflammatory cytokines, such as TNF-α, IL-17A, and IFN-γ in UC patients. Therefore, we further detected these three pro-inflammatory factors in the mouse model. ELISA showed that the levels of TNF-α (*P* = 0.0005, *P* = 0.0005), IL-17 (*P* = 0.0014, *P* = 0.0381), and IFN-γ (*P* = 0.0016, *P* = 0.0042) were prominently increased in the serum and colonic tissue of the DSS group compared with the control mice (Fig. 7A–F).

## Discussion

UC is characterized by chronic inflammation, a relapsing and intermittent course, and a substantial risk of colorectal cancer [2, 32, 33]. The pathogenesis of UC remains unclear. However, accumulating evidence has suggested that epigenetic mechanisms, such as DNA methylation, are contributing factors in the pathogenesis of UC [34–36]. Moreover, hyper-methylation or hypo-methylation with corresponding down-regulation or up-regulation of the associated genes has been observed in UC [7, 9, 37]. Wetzel et al. have reported that under inflammatory and demethylating conditions, the upregulation of *Epstein–Barr virus-induced gene* 3 (EBI3) contributes to the formation of anti-inflammatory IL-35, which may be considered a therapeutic target in UC [38]. In the present study, we analyzed the DNA methylation of GSE27899 in the GEO database and found 48 differential genes with significant changes in methylation. Among them, the methylation level of Zbtb7b was significantly higher compared with the other differential genes in the HC group. Moreover, we found that the Zbtb7b was demethylated and up-regulated in UC samples compared with HC samples. In addition, the expression of Zbtb7b was positively associated with the degree of UC activity. Therefore, ZBTB7B might be a diagnostic and therapeutic biomarker for UC.

Zbtb7b is a transcription factor of a zinc finger and BTB domain-containing protein 7B, which is also called T-helper-inducing POZ/Krueppel-like factor (ThPOK), and it is primarily involved in the differentiation and development of T cells and NK T cells [39, 40]. Dave et al*.* have found that when spontaneous helper T cell deletion (HD) occurs in mice, CD4^+^ T cell differentiation is impaired, and CD4^+^ T cells in peripheral blood are deleted [41]. Moreover, Zbtb7b is the gene of HD deletion. The point mutation of the Zbtb7b gene in HD mice leads to the change of zinc finger domain residues of ZBTB7B protein binding to DNA, making ZBTB7B unable to bind to target DNA and leading to an HD phenotype [42]. Wang et al. have found that Zbtb7b plays a vital role in activating the maturation of peripheral CD4^+^T cells, leading to repressed differentiation of peripheral CD8^+^T cells [28]. It has been discovered that Zbtb7b-deficient mice lack CD4^+^T cells but harbor a population of CD8^+^T cells in peripheral blood and spleen [43, 44]. Kennedy et al. have also reported that Zbtb7b-deficient mice experience a defect in T cell maturation, contributing to dramatically decreased numbers of peripheral CD4^+^T cells and subsequently reduced production of pro-inflammatory cytokines [45]. In recent years, the role of Zbtb7b in intestinal immune function has been gradually expanded. In the intestinal environment, Zbtb7b and Runt-related transcription factor 3 (RUNX3) antagonize each other. The former inhibits the cytotoxic function of CD4^+^ intraepithelial T lymphocytes (IELs) and maintains the inflammatory function of CD4^+^ IELs. When Zbtb7b is absent, the activated CD4^+^ IELs lose their inflammatory function, resulting in the inability to induce colitis. The expressions of Zbtb7b and RUNX3 restrict each other, affect the response of CD4^+^ T cells, and impair intestinal inflammatory immunity and the probability of exogenous bacterial infection [15–17]. In conclusion, previous studies have shown that Zbtb7b is related to the differentiation of CD4^+^ T cells. Inhibition or deletion of Zbtb7b will mediate the reduction of the intestinal immune response, resulting in less colitis. In the present study, we also found that Zbtb7b was significantly up-regulated in colonic tissue samples from UC patients and the DSS-induced colitis model.

Meanwhile, several CD4^+^ T cells were harbored in the peripheral blood and inflamed colonic tissues. Therefore, we considered that the expression of Zbtb7b was associated with the disease severity of UC and might function as a possible target for the management of UC via regulating the differentiation of CD4^+^ T cells. Yang et al. have shown that the activation of CD4^+^ T cells can significantly elevate the expressions of inflammatory cytokines, such as TNF-α, IL-17A, and IFN-γ, in peripheral blood of UC patients [31]. Another study has reported that the activation of CD4^+^ T cells predominantly produces inflammatory cytokines of TNF-α, IL-17, and IFN-γ in IBDs [46]. In the present study, we found that the inflammatory cytokines of TNF-α, IL-17, and IFN-γ were up-regulated in the DSS-induced colitis model. Therefore, we considered that the over-expression of Zbtb7b promoted the differentiation of CD4^+^T cells, subsequently contributing to the production of inflammatory cytokines in the DSS-induced colitis model.

Barragan et al. have revealed that *Lactobacillus reuters* provide indole-derivatives, such as indole-3-lactic acid, that activate the aryl-hydrocarbon receptor (AhR) and contribute to the down-regulation of Zbtb7b in IELs, which repress the differentiation of CD4^+^ T and activate the differentiation of CD8^+^ T cells, resulting in reprogramming of CD4^+^ T cells into DP CD4^+^CD8^+^ T cells [47]. Furthermore, DP CD4^+^CD8^+^ T cells can maintain a steady-state of the gut mucosa and inhibit pro-inflammatory cytokine release during pathogenic infection [47]. Das et al. have shown that DP CD4^+^CD8^+^ T cells reside in the intestinal epithelial layer and possess a regulatory function in inhibiting type 1 helper T (Th1) cell-induced intestinal inflammation [48]. In the present study, we found that the proportion of DP CD4^+^CD8^+^ T cells was significantly decreased in the inflamed colonic tissues in the DSS-induced colitis model. Therefore, over-expression of Zbtb7b might repress the differentiation of DP CD4^+^CD8^+^ T cells and then increase the production of inflammatory cytokines, such as TNF-α, IL-17, and IFN-γ, in the DSS-induced colitis model.

However, there were several limitations in our study. First, due to the amount of tissue and detection technology, we failed to detect the methylation of Zbtb7b in fresh intestinal mucosa from UC patients and HCs. In addition, we initially explored the relationship between demethylated Zbtb7b and T cell immunity in the DSS-induced colitis model, while those findings were not further verified in vitro. Finally, we found the hypo-methylation of Zbtb7b through the GEO database and preliminary verified it in the mouse model of colitis. The hypo-methylated Zbtb7b might be related to the regulation of Zbtb7b as the maturation of CD4^+^ T cells and the differentiation of DP CD4^+^CD8^+^ T cells, resulting in the production of inflammatory cytokines and colonic inflammation. However, further research and more data support are still necessary.

**Fig. 8:**
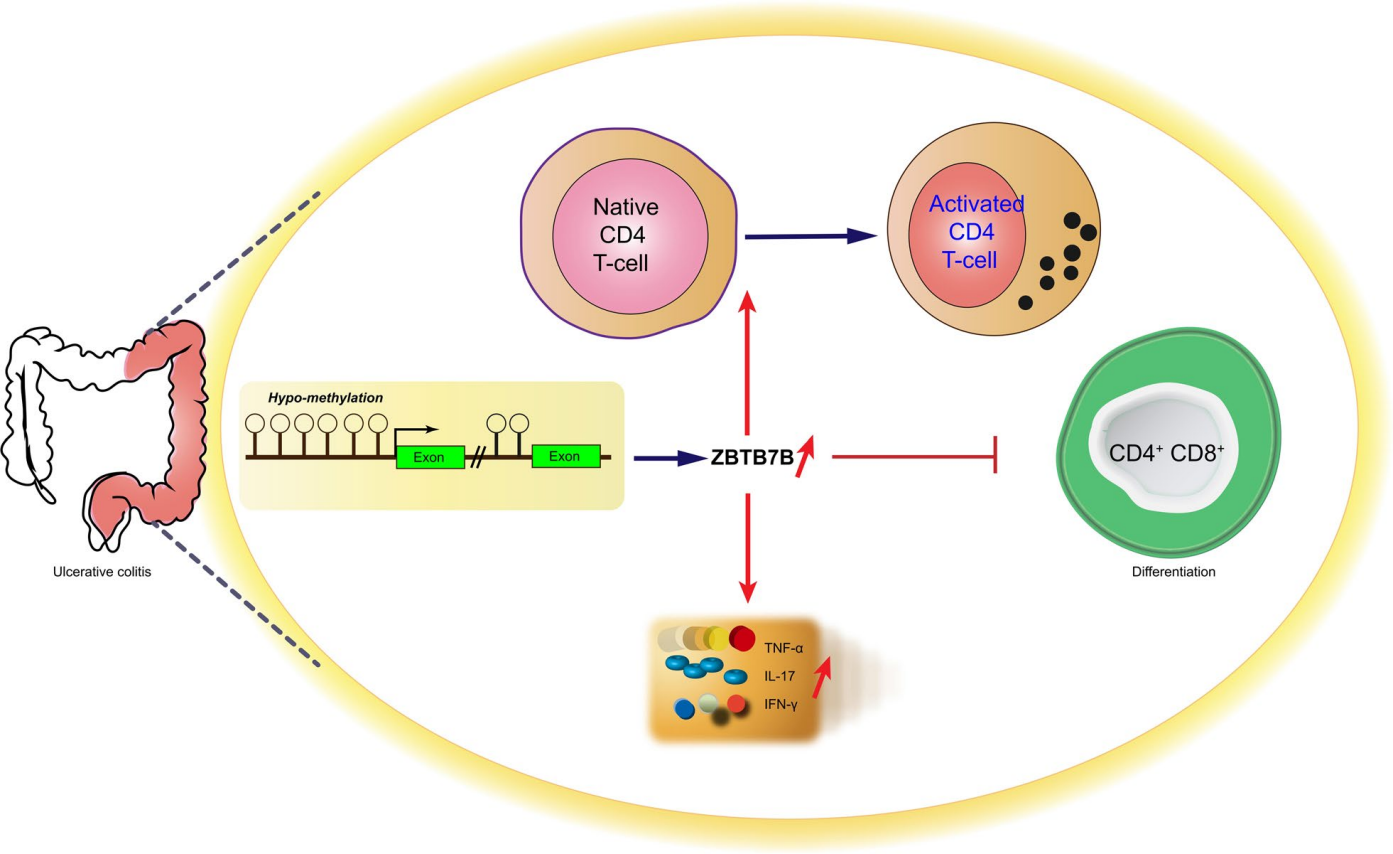
Proposed mechanism of demethylated Zbtb7b affecting the pathogenesis of UC by regulating intestinal immunity. Hypo-methylation plays an important role in the pathogenesis of UC, and Zbtb7b was demethylated and up-regulated in the colonic tissues of UC patients. Furthermore, demethylated Zbtb7b activated the maturation of CD4^+^T cells and repressed the differentiation of DP CD4^+^CD8^+^ T cells, resulting in the production of inflammatory cytokines (TNF-α, IL-17, and IFN-γ) and colonic inflammation in UC

In summary, we demonstrated that Zbtb7b was demethylated and up-regulated in the UC and DSS-induced colitis model. Moreover, over-expression of Zbtb7b activated the maturation of CD4^+^T cells and repressed the differentiation of DP CD4^+^CD8^+^ T cells, which contributed to the production of inflammatory cytokines, such as TNF-α, IL-17, and IFN-γ, in the DSS-induced colitis model (Fig. 8). Therefore, Zbtb7b might be a diagnostic and therapeutic biomarker for UC, and hypo-methylation might affect the biological function of Zbtb7b.

## Conclusions

In the present study, we demonstrated that Zbtb7b was related to the pathogenesis of UC by bioinformatics analysis. We further verified that demethylated Zbtb7b activated the maturation of CD4^+^T cells and repressed the differentiation of DP CD4^+^CD8^+^ T cells, resulting in the production of inflammatory cytokines and colonic inflammation in the UC and DSS-induced colitis model. Therefore, Zbtb7b might be a diagnostic and therapeutic biomarker for UC, and hypo-methylation might affect the biological function of Zbtb7b.

## Supplementary Information

**Additional file 1:** **Fig. S1. **WB of ZBTB7B in colonic tissue of mice. WB was used to detect ZBTB7B protein content in colonic tissue of mice. NC: normal control group mouse, n = 5; DSS: DSS-induced colitis group mouse, n = 7.

**Additional file 2:** **Fig. S2. **Specific steps of detecting CD4/CD8 T cells in mouse colonic tissue by FCM. Step 1: Circling lymphocytes: circled the lymphocyte population with FSC and SSC according to the cell size and granularity; Step 2–4: Removing the adhesive: circled the single cells without adhesions according to the fluorescence area and height of laterally scattered light. Then, circled the single cells without adhesions according to the fluorescent area and height of forward scattered light; Step 5: Circling living cells: marked dead and living cells with Zombie Yellow. Dead cells were marked and expressed as positive, while living cells could not be marked and expressed as negative; Step 6: Circling white blood cells: white blood cells were labeled with anti-CD45 antibody and obviously clustered; Step 7: Circling T cells: T cells were labeled with anti-CD3 antibody and obviously clustered; Step 8: Circle CD4^+^/CD8^+^ T cells: CD4^+^/CD8^+^ T cells were labeled with anti-CD4 antibody/anti-CD8 antibody respectively, and the cells in the quadrant with positive expression were DP CD4 + CD8 + T cells.

**Additional file 3: Table S1. **The demographic and clinical characteristics of 15 UC patients.

## Data Availability

The datasets generated during and/or analyzed during the current study are available in the Gene Expression Omnibus (GEO) datasets (http://www.ncbi.nlm.nih.gov/geo/). Other datasets used and analyzed during the current study are available from the corresponding authors on reasonable request.

## Abbreviations

UC: Ulcerative colitis
Zbtb7b/ZBTB7B: Zinc finger and BTB domain-containing protein 7B
ThPOK: Th-inducing POZ-Kruppel factor
IBD: Inflammatory bowel diseases
GEO: Gene expression omnibus
DSS: Dextran sodium sulfate
DEGs: Differentially expressed genes
KEGG: Kyoto encyclopedia of genes and genomes
qRT-PCR: Quantitative real-time PCR
WB: Western blot
IHC: Immunohistochemistry
DAI: Disease activity index
FCM: Flow cytometry
HE: Hematoxylin–eosin
IF: Immunofluorescence
MSP: Methylation-specific PCR
ELISA: Enzyme-linked immunosorbent assay
mAbs: Monoclonal antibodies
AF: Alexa Fluor
FITC: Flurescein isothiocyanate
PerCP: Peridinin-chlorophyll-protein
BV: Brilliant violet
SD: Standard deviation
SEM: Standard error of mean
ANOVA: Analysis of variance
NK: Natural killer
HC: Healthy control
NC: Normal control
EBI3: Epstein–Barr virus induced gene 3
AhR: Aryl-hydrocarbon receptor
IELs: Intraepithelial T lymphocytes
DP CD4^+^CD8^+^ T: Double positive CD4^+^CD8^+^ T cells
Th1: Type 1 helper T
RUNX3: Runt related transcription factor 3
HD: Helper T cell deletion

## References

[1] Graham DB, Xavier RJ (2020). Pathway paradigms revealed from the genetics of inflammatory bowel disease. Nature.

[2] Chang JT (2020). Pathophysiology of inflammatory bowel diseases. N Engl J Med.

[3] Ng SC, Shi HY, Hamidi N, Underwood FE, Tang W, Benchimol EI, Panaccione R, Ghosh S, Wu JCY, Chan FKL (2017). Worldwide incidence and prevalence of inflammatory bowel disease in the 21st century: a systematic review of population-based studies. Lancet.

[4] Ng SC, Kaplan GG, Tang W, Banerjee R, Adigopula B, Underwood FE, Tanyingoh D, Wei SC, Lin WC, Lin HH (2019). Population density and risk of inflammatory bowel disease: a prospective population-based study in 13 countries or regions in Asia-Pacific. Am J Gastroenterol.

[5] Kaur A, Goggolidou P (2020). Ulcerative colitis: understanding its cellular pathology could provide insights into novel therapies. J Inflamm (Lond).

[6] Jostins L, Ripke S, Weersma RK, Duerr RH, McGovern DP, Hui KY, Lee JC, Schumm LP, Sharma Y, Anderson CA (2012). Host–microbe interactions have shaped the genetic architecture of inflammatory bowel disease. Nature.

[7] McDermott E, Ryan EJ, Tosetto M, Gibson D, Burrage J, Keegan D, Byrne K, Crowe E, Sexton G, Malone K (2016). DNA methylation profiling in inflammatory bowel disease provides new insights into disease pathogenesis. J Crohns Colitis.

[8] Murphy TM, Mill J (2014). Epigenetics in health and disease: heralding the EWAS era. Lancet.

[9] Tahara T, Hirata I, Nakano N, Nagasaka M, Nakagawa Y, Shibata T, Ohmiya N (2017). Comprehensive DNA methylation profiling of inflammatory mucosa in ulcerative colitis. Inflamm Bowel Dis.

[10] Taman H, Fenton CG, Hensel IV, Anderssen E, Florholmen J, Paulssen RH (2018). Genome-wide DNA methylation in treatment-naive ulcerative colitis. J Crohns Colitis.

[11] Nimmo ER, Prendergast JG, Aldhous MC, Kennedy NA, Henderson P, Drummond HE, Ramsahoye BH, Wilson DC, Semple CA, Satsangi J (2012). Genome-wide methylation profiling in Crohn's disease identifies altered epigenetic regulation of key host defense mechanisms including the Th17 pathway. Inflamm Bowel Dis.

[12] Cooke J, Zhang H, Greger L, Silva AL, Massey D, Dawson C, Metz A, Ibrahim A, Parkes M (2012). Mucosal genome-wide methylation changes in inflammatory bowel disease. Inflamm Bowel Dis.

[13] Hasler R, Feng Z, Backdahl L, Spehlmann ME, Franke A, Teschendorff A, Rakyan VK, Down TA, Wilson GA, Feber A (2012). A functional methylome map of ulcerative colitis. Genome Res.

[14] Kraiczy J, Nayak K, Ross A, Raine T, Mak TN, Gasparetto M, Cario E, Rakyan V, Heuschkel R, Zilbauer M (2016). Assessing DNA methylation in the developing human intestinal epithelium: potential link to inflammatory bowel disease. Mucosal Immunol.

[15] Reis BS, Rogoz A, Costa-Pinto FA, Taniuchi I, Mucida D (2013). Mutual expression of the transcription factors Runx3 and ThPOK regulates intestinal CD4(+) T cell immunity. Nat Immunol.

[16] Reis BS, Hoytema van Konijnenburg DP, Grivennikov SI, Mucida D (2014). Transcription factor T-bet regulates intraepithelial lymphocyte functional maturation. Immunity.

[17] Park Y, Moon SJ, Lee SW (2016). Lineage re-commitment of CD4CD8alphaalpha intraepithelial lymphocytes in the gut. BMB Rep.

[18] Wirtz S, Neufert C, Weigmann B, Neurath MF (2007). Chemically induced mouse models of intestinal inflammation. Nat Protoc.

[19] Murthy SN, Cooper HS, Shim H, Shah RS, Ibrahim SA, Sedergran DJ (1993). Treatment of dextran sulfate sodium-induced murine colitis by intracolonic cyclosporin. Dig Dis Sci.

[20] Lin H, Wang Q, Liu L, Chen Z, Das R, Zhao Y, Mao D, Luo Y (2020). Colonization of mice with amoxicillin-associated *Klebsiella variicola* drives inflammation via th1 induction and treg inhibition. Front Microbiol.

[21] Hughes S, Jones JL (2007). The use of multiple displacement amplified DNA as a control for methylation specific PCR, pyrosequencing, bisulfite sequencing and methylation-sensitive restriction enzyme PCR. BMC Mol Biol.

[22] Russo AL, Thiagalingam A, Pan H, Califano J, Cheng KH, Ponte JF, Chinnappan D, Nemani P, Sidransky D, Thiagalingam S (2005). Differential DNA hypermethylation of critical genes mediates the stage-specific tobacco smoke-induced neoplastic progression of lung cancer. Clin Cancer Res.

[23] Zhao C, Qiu S, He J, Peng Y, Xu H, Feng Z, Huang H, Du Y, Zhou Y, Nie Y (2020). Prodigiosin impairs autophagosome-lysosome fusion that sensitizes colorectal cancer cells to 5-fluorouracil-induced cell death. Cancer Lett.

[24] Lu L, Qin A, Huang H, Zhou P, Zhang C, Liu N, Li S, Wen G, Zhang C, Dong W (2011). Shikonin extracted from medicinal Chinese herbs exerts anti-inflammatory effect via proteasome inhibition. Eur J Pharmacol.

[25] Shi X, Chen X, Li X, Lan X, Zhao C, Liu S, Huang H, Liu N, Liao S, Song W (2014). Gambogic acid induces apoptosis in imatinib-resistant chronic myeloid leukemia cells via inducing proteasome inhibition and caspase-dependent Bcr-Abl downregulation. Clin Cancer Res.

[26] Chow J, Hoffend NC, Abrams SI, Schwaab T, Singh AK, Muhitch JB (2020). Radiation induces dynamic changes to the T cell repertoire in renal cell carcinoma patients. Proc Natl Acad Sci USA.

[27] Sargent PB (1994). Double-label immunofluorescence with the laser scanning confocal microscope using cyanine dyes. Neuroimage.

[28] Wang L, Wildt KF, Castro E, Xiong Y, Feigenbaum L, Tessarollo L, Bosselut R (2008). The zinc finger transcription factor Zbtb7b represses CD8-lineage gene expression in peripheral CD4+ T cells. Immunity.

[29] Cheroutre H, Husain MM (2013). CD4 CTL: living up to the challenge. Semin Immunol.

[30] Lee HO, He X, Mookerjee-Basu J, Zhongping D, Hua X, Nicolas E, Sulis ML, Ferrando AA, Testa JR, Kappes DJ (2015). Disregulated expression of the transcription factor ThPOK during T-cell development leads to high incidence of T-cell lymphomas. Proc Natl Acad Sci USA.

[31] Yang Y, Zhang C, Jing D, He H, Li X, Wang Y, Qin Y, Xiao X, Xiong H, Zhou G (2021). IRF5 acts as a potential therapeutic marker in inflammatory bowel diseases. Inflamm Bowel Dis.

[32] Tang B, Zhu J, Fang S, Wang Y, Vinothkumar R, Li M, Weng Q, Zheng L, Yang Y, Qiu R (2021). Pharmacological inhibition of MELK restricts ferroptosis and the inflammatory response in colitis and colitis-propelled carcinogenesis. Free Radic Biol Med.

[33] Sugita A, Ikeuchi H, Funayama Y, Futami K, Iiai T, Itabashi M, Suzuki Y (2021). Postoperative survival in colitis-associated colorectal cancer with ulcerative colitis in Japan: a multicenter analysis. Anticancer Res.

[34] Gould NJ, Davidson KL, Nwokolo CU, Arasaradnam RP (2016). A systematic review of the role of DNA methylation on inflammatory genes in ulcerative colitis. Epigenomics.

[35] Su S, Kong W, Zhang J, Wang X, Guo H. Integrated analysis of DNA methylation and gene expression profiles identified S100A9 as a potential biomarker in ulcerative colitis. Biosci Rep. 2020;40(12). 10.1042/BSR20202384 PMC7711060 33185247

[36] Rosa I, Silva P, da Mata S, Magro F, Carneiro F, Peixoto A, Silva M, Sousa HT, Roseira J, Parra J (2020). Methylation patterns in dysplasia in inflammatory bowel disease patients. Scand J Gastroenterol.

[37] Taman H, Fenton CG, Anderssen E, Florholmen J, Paulssen RH (2021). DNA hypo-methylation facilitates anti-inflammatory responses in severe ulcerative colitis. PLoS ONE.

[38] Wetzel A, Scholtka B, Schumacher F, Rawel H, Geisendorfer B, Kleuser B. Epigenetic DNA methylation of EBI3 modulates human interleukin-35 formation via NFkB signaling: a promising therapeutic option in ulcerative colitis. Int J Mol Sci. 2021;22(10). 10.3390/ijms22105329 PMC8158689 34069352

[39] Muroi S, Naoe Y, Miyamoto C, Akiyama K, Ikawa T, Masuda K, Kawamoto H, Taniuchi I (2008). Cascading suppression of transcriptional silencers by ThPOK seals helper T cell fate. Nat Immunol.

[40] Enders A, Stankovic S, Teh C, Uldrich AP, Yabas M, Juelich T, Altin JA, Frankenreiter S, Bergmann H, Roots CM (2012). ZBTB7B (Th-POK) regulates the development of IL-17-producing CD1d-restricted mouse NKT cells. J Immunol.

[41] Dave VP, Allman D, Keefe R, Hardy RR, Kappes DJ (1998). HD mice: a novel mouse mutant with a specific defect in the generation of CD4(+) T cells. Proc Natl Acad Sci USA.

[42] He X, He X, Dave VP, Zhang Y, Hua X, Nicolas E, Xu W, Roe BA, Kappes DJ (2005). The zinc finger transcription factor Th-POK regulates CD4 versus CD8 T-cell lineage commitment. Nature.

[43] Engel I, Hammond K, Sullivan BA, He X, Taniuchi I, Kappes D, Kronenberg M (2010). Co-receptor choice by V alpha14i NKT cells is driven by Th-POK expression rather than avoidance of CD8-mediated negative selection. J Exp Med.

[44] Wang L, Carr T, Xiong Y, Wildt KF, Zhu J, Feigenbaum L, Bendelac A, Bosselut R (2010). The sequential activity of Gata3 and Thpok is required for the differentiation of CD1d-restricted CD4+ NKT cells. Eur J Immunol.

[45] Kennedy JM, Georges A, Bassenden AV, Vidal SM, Berghuis AM, Taniuchi I, Majewski J, Lathrop M, Behr MA, Langlais D, et al. ZBTB7B (ThPOK) is required for pathogenesis of cerebral malaria and protection against pulmonary tuberculosis. Infect Immun. 2020;88(2). 10.1128/IAI.00845-19 PMC6977123 31792077

[46] Mickael ME, Bhaumik S, Basu R (2020). Retinoid-related orphan receptor RORgammat in CD4(+) T-cell-mediated intestinal homeostasis and inflammation. Am J Pathol.

[47] Cervantes-Barragan L, Chai JN, Tianero MD, Di Luccia B, Ahern PP, Merriman J, Cortez VS, Caparon MG, Donia MS, Gilfillan S (2017). Lactobacillus reuteri induces gut intraepithelial CD4(+)CD8alphaalpha(+) T cells. Science.

[48] Das G, Augustine MM, Das J, Bottomly K, Ray P, Ray A (2003). An important regulatory role for CD4+CD8 alpha alpha T cells in the intestinal epithelial layer in the prevention of inflammatory bowel disease. Proc Natl Acad Sci USA.

