# Supplementary figures and images for: Epigenetic DNA methylation of Zbtb7b regulates the population of double-positive CD4+CD8+ T cells in ulcerative colitis
Source: J Transl Med. 2022 Jun 27;20:289. doi: 10.1186/s12967-022-03477-6 (PMC9235105; doi:10.1186/s12967-022-03477-6)

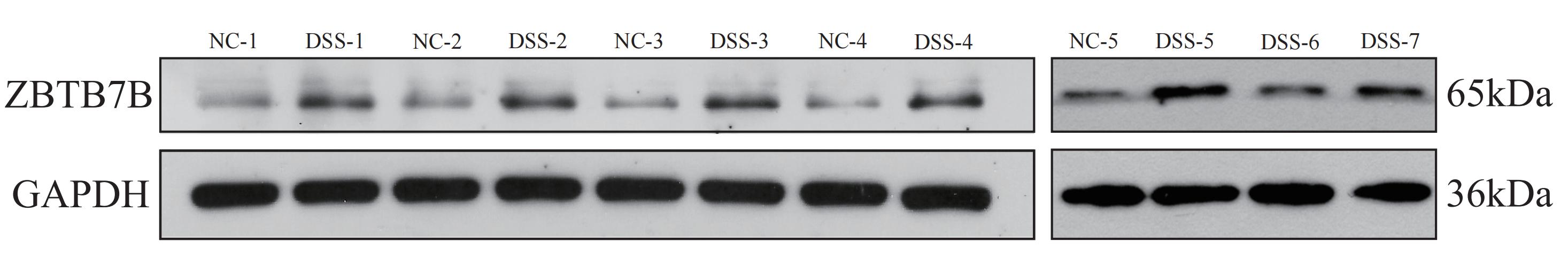

Supplement: Supplementary file 1 — Additional file 1: Fig. S1. WB of ZBTB7B in colonic tissue of mice. WB was used to detect ZBTB7B protein content in colonic tissue of mice. NC: normal control group mouse, n = 5; DSS: DSS-induced colitis group mouse, n = 7. [file 12967_2022_3477_MOESM1_ESM.jpg]

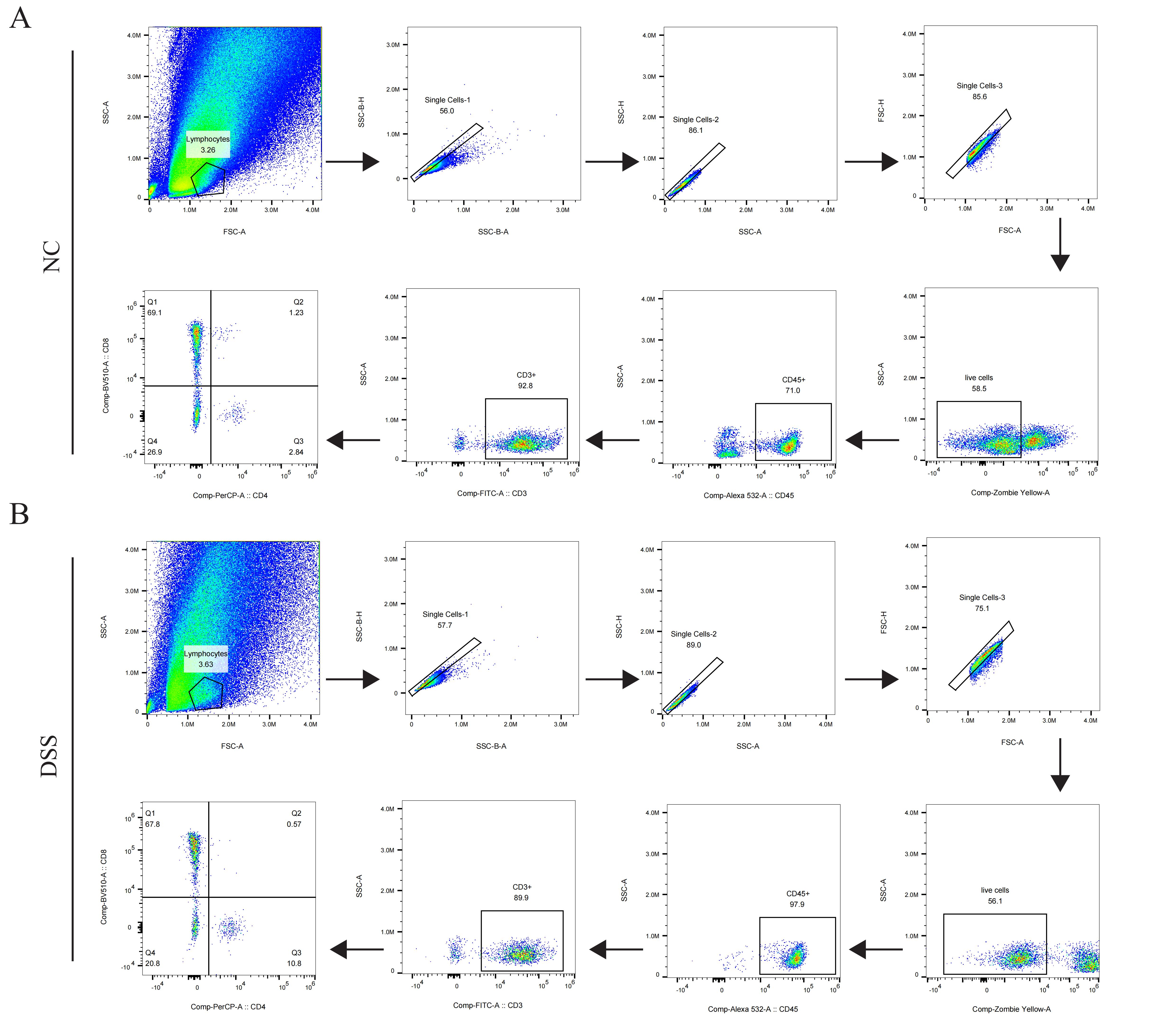

Supplement: Supplementary file 2 — Additional file 2: Fig. S2. Specific steps of detecting CD4/CD8 T cells in mouse colonic tissue by FCM. Step 1: Circling lymphocytes: circled the lymphocyte population with FSC and SSC according to the cell size and granularity; Step 2–4: Removing the adhesive: circled the single cells without adhesions according to the fluorescence area and height of laterally scattered light. Then, circled the single cells without adhesions according to the fluorescent area and height of forward scattered light; Step 5: Circling living cells: marked dead and living cells with Zombie Yellow. Dead cells were marked and expressed as positive, while living cells could not be marked and expressed as negative; Step 6: Circling white blood cells: white blood cells were labeled with anti-CD45 antibody and obviously clustered; Step 7: Circling T cells: T cells were labeled with anti-CD3 antibody and obviously clustered; Step 8: Circle CD4+/CD8+ T cells: CD4+/CD8+ T cells were labeled with anti-CD4 antibody/anti-CD8 antibody respectively, and the cells in the quadrant with positive expression were DP CD4 + CD8 + T cells. [file 12967_2022_3477_MOESM2_ESM.jpg]
