# Supplementary material for: Epigenetic DNA methylation of Zbtb7b regulates the population of double-positive CD4+CD8+ T cells in ulcerative colitis
Source: J Transl Med. 2022 Jun 27;20:289. doi: 10.1186/s12967-022-03477-6 (PMC9235105; doi:10.1186/s12967-022-03477-6)
Supplement: Supplementary file 3 — Additional file 3: Table S1. The demographic and clinical characteristics of 15 UC patients. [file 12967_2022_3477_MOESM3_ESM.docx]

| **Table S1. The demographic and clinical characteristics of 15 UC patients** | | | | | |
| --- | --- | --- | --- | --- | --- |
| Patient ID | Sex | Age (years) | Montreal type | Total Mayo scores | Items |
| 1 | M | 35 | E1 | 4 | IHC |
| 2 | F | 26 | E1 | 5 | IHC |
| 3 | M | 34 | E2 | 7 | IHC |
| 4 | F | 49 | E1 | 5 | IHC |
| 5 | M | 52 | E1 | 6 | IHC |
| 6 | M | 45 | E3 | 8 | IHC |
| 7 | F | 45 | E1 | 5 | IHC |
| 8 | F | 62 | E2 | 7 | IHC |
| 9 | M | 46 | E1 | 6 | IHC |
| 10 | F | 47 | E2 | 6 | IHC |
| 11 | M | 33 | E2 | 8 | WB |
| 12 | F | 41 | E2 | 8 | WB |
| 13 | M | 54 | E2 | 6 | WB |
| 14 | F | 49 | E1 | 6 | WB |
| 15 | M | 36 | E1 | 6 | WB |

Notes: The nomal controls were age - and sex - matched healthy volunteers, and the relevant information was not listed. F: female; M: male; IHC: immunohistochemistry; WB: western blot.
